# How Does Nostalgia Conduce to Global Self-Continuity? The Roles of Identity Narrative, Associative Links, and Stability

**DOI:** 10.1177/01461672211024889

**Published:** 2021-06-21

**Authors:** Emily K. Hong, Constantine Sedikides, Tim Wildschut

**Affiliations:** 1 University of Southampton, UK

**Keywords:** nostalgia, self-continuity, narrative, associative links, stability

## Abstract

In five studies (*N* = 1,074), we examined the relation—both correlational and causal—between nostalgia, a sentimental longing for one’s past, and global self-continuity (GSC), a sense of connection among past, present, and future selves. Furthermore, we addressed mechanisms underlying this relation. We asked, in particular, whether nostalgic individuals might achieve GSC by constructing a narrative to give meaning to life transitions (narrative), connecting to the past (associative links), or believing in a self that is resistant to change (stability). Nostalgia predicted (Studies 1–3) and caused (Studies 4 and 5) GSC. The relation between nostalgia and GSC was consistently mediated by narrative, sporadically mediated by associative links, and unmediated by stability. The robust indirect effect via narrative remained significant when controlling for rumination (Study 3). We discuss theoretical and practical implications.

Nostalgia, a sentimental longing for one’s past, enables mental time travel. Nostalgic reverie makes the present self feel more tethered to the past self. But does it also make the present self feel more tethered both to the past and future selves? We are concerned with the relation—naturalistic and causal—between nostalgia and self-continuity and with the mechanisms underlying this relation.

## The Profile of Nostalgia

Nostalgia can be evoked incidentally (e.g., through music, songs, scents, or tastes) or deliberately (e.g., by engaging in reflection or conversing with friends; Sedikides et al., 2015b). Nostalgic accounts comprise, for the most part, unique or infrequent life events (e.g., birthday celebrations, family vacations, anniversaries, and graduations), keepsakes, or close others (e.g., partners, friends, and family members; Abeyta et al., 2015; Wildschut et al., 2006). In nostalgizing, the individual feels warm, tender, and contented but tinged with yearning and some sadness for the irredeemably bygone past (Hepper et al., 2012, 2014). Nostalgia, therefore, is an ambivalent, but predominantly positive, emotion (Leunissen et al., 2021; Sedikides & Wildschut, 2016a). Furthermore, nostalgia is self-relevant: The individual is the protagonist of personally meaningful nostalgic occasions (Sedikides & Wildschut, 2018; Van Tilburg et al., 2018). It is also social: close others play a key or supportive role in nostalgic accounts, and nostalgia is associated with or increases social connectedness (i.e., a sense of belongingness and acceptance; Frankenbach et al., 2021; Sedikides & Wildschut, 2019). Taken together, nostalgia is a frequently experienced emotion (i.e., several times a week; Wildschut et al., 2006) that transpires across ages (Hepper et al., 2021) and cultures (Hepper et al., 2014).

Nostalgia is related to and augments approach motivation (Juhl et al., 2021; Stephan et al., 2014), which orients the individual toward the future. Nostalgia is also related to and elevates optimism (Cheung et al., 2013; Kersten et al., 2016). Moreover, nostalgia increases openness to experience (Hotchin & West, 2021; Van Tilburg et al., 2015), strengthens intentions to seek out future opportunities for psychological growth (Baldwin & Landau, 2014; Biskas et al., 2019), and galvanizes the resolve to pursue one’s important goals (Sedikides et al., 2018). In the same vein, nostalgia nurtures a sense of youthfulness (i.e., lower subjective age, alertness or energy; Abeyta & Routledge, 2016), heightens inspiration (i.e., transcendence of mundane preoccupations, awareness of opportunities; Stephan et al., 2015), and enhances financial risk-taking (Zou et al., 2019).

If nostalgia involves mental time travel to the past (Evans et al., 2021; Watts et al., 2020), does it conduce to or foster a sense of connection between one’s present and past selves—what we label present-to-past self-continuity? Moreover, if nostalgia involves mental time travel to the future (FioRito & Routledge, 2020; Salmon & Wohl, 2020), does it conduce to or foster a sense of connection among one’s past, present, and future selves—what we label global self-continuity (GSC)?

## Nostalgia and Self-Continuity

### Nostalgia and Present-to-Past Self-Continuity

Traditional theorizing concerns present-to-past self**-**continuity. William James (1890) pioneered its definition and speculated on its psychological construction: The “I” (observer or self as knower) perceives “Me” (the actor or self as object) across time and evaluates whether the actors are coherent enough at various temporal points to be unified as a single observer. So, continuity is a key attribute of the “I.” Other philosophers concurred that continuity in the midst of psychological change is a prerequisite of identity (Madell, 1981; Taylor, 1989; Wiggins, 2001) as did psychologists (Atchley, 1989; Erikson, 1968; Neisser, 1988). Psychologists additionally advocated a more mainstream positioning of the construct (Breakwell, 1986; Habermas & Bluck, 2000), pointing to the human need to achieve and sustain self-continuity (Vignoles, 2011; Vignoles et al., 2006). Its pursuit is functional. Self-continuity is prognostic of positive affect, psychological well-being (McAdams et al., 2001; Troll & Skaff, 1997), and psychological equanimity (Landau et al., 2008, 2009) as well as decreased negative affect, anxiety, and psychopathology (Chandler et al., 2003; Lampinen et al., 2004).

Davis (1979) was the first to theorize a link between nostalgia and present-to-past self**-**continuity when he stated that the emotion “marshal[s] our psychological resources for continuity” (p. 34). A correlational study (Zou et al., 2018) indeed found that nostalgia for one’s home country and nostalgia for one’s host country following repatriation, each assessed in reference to 10 objects (e.g., “my family” and “my friends,”), were positively related to present-to-past self**-**continuity (i.e., “I feel connected with my past” and “I feel there is continuity in my life”).

Experimental research further supported Davis’ (1979) intuition, establishing a causal relation between nostalgia and present-to-past self**-**continuity. In the first relevant experiment (Sedikides et al., 2015a, Experiment 3), nostalgia was induced with the Event Reflection Task (Sedikides et al., 2015b; Wildschut et al., 2006). Participants in the nostalgia condition reflected on a nostalgic event in their lives, listed a few keywords about it, and then described the event in writing. Participants in the control condition followed the same protocol but for an ordinary event in their lives. Then, all participants indicated the extent to which they experienced self-continuity (i.e., “I feel connected with my past,” “I feel connected with who I was in the past,” “There is continuity in my life,” and “Important aspects of my personality remain the same across time”). Nostalgic participants reported higher present-to-past self-continuity than controls. This finding has been replicated in various countries (i.e., China, Greece, Syria, United Kingdom, United States; Abakoumkin et al., 2019; Sedikides et al., 2016c; Van Tilburg et al., 2019b; Wildschut et al., 2019), with alternative inductions of the emotion (e.g., based on the cognitive prototype of the construct “nostalgia”—Abakoumkin et al., 2019, Experiment 2; based on song lyrics—Sedikides et al., 2016c, Experiment 1), with alternative control conditions (e.g., reflecting on positive memories from one’s past—Sedikides et al., 2015a, Experiment 4; Sedikides et al., 2016c, Experiment 3), and with both non-student and non-online platform samples (i.e., Syrian refugees—Wildschut et al., 2019; people living with dementia—Ismail et al., 2018).

### Nostalgia and GSC

The conceptualization of self-continuity as a connection among one’s past, present, and future selves also has a history in philosophy (Lewis, 1976; Parfit, 1971; Strawson, 2009) and psychology (Chandler, 1994; Rutt & Löckenhoff, 2016; Sani, 2008). Indeed, present-to-past and present-to-future self-continuity are positively related (Sokol & Eisenheim, 2016). Furthermore, psychologists have pointed to the human need to attain and maintain GSC (Vignoles, 2011). Its pursuit is functional. GSC is prognostic of higher positive affect and satisfaction with life (Sokol & Serper, 2019), adaptive (i.e., more rational and detached) coping (Sadeh & Karniol, 2012), as well as social well-being (i.e., actualization, coherence, social acceptance, contribution, and integration into one’s community; Sani et al., 2008) and collective self-esteem (Sani et al., 2007).

As mentioned, nostalgia entails mental time travel not only to one’s past but also to one’s future (Sedikides & Wildschut, 2016b, 2020). Put otherwise, although nostalgia is a past-oriented emotion, it has implications for one’s future. One uses nostalgic reverie to align their past identity to the future one (e.g., “I have been caring and will continue to be so”) or derive inspiration for it (e.g., “My childhood means a lot to me, and I want my children to feel the same about their own”). In addition, nostalgia, as a positive emotion, may encourage a broader outlook on one’s life (Fredrickson, 2001), bridging the present with the future. Nostalgia may be linked to GSC.

## The Current Research

We addressed the relation—correlational and causal—between nostalgia and GSC. More importantly, we investigated the underlying mechanisms.

### Mediators of the Relation Between Nostalgia and GSC

We explored three mechanisms through which nostalgia might relate to or cause GSC. We derived these mechanisms (i.e., bases of GSC) from a cross-cultural study (Becker et al., 2018, p. 283), where participants first generated eight answers (i.e., identity aspects) to the question “Who are you?” and then indicated the extent to which each identity aspect made them “feel that your past, present, and future are connected” (i.e., contributed to GSC). Subsequently, participants responded to measures of three putative bases of GSC: narrative (whether each identity aspect depicts one’s life as a story), associative links (whether each identity aspect reminds one of their past), and stability (whether each identity aspect is stable). We note that these three questions invited corresponding judgments or appraisals: a sense of narrative, associative links, and stability. Each identity aspect or basis positively predicted GSC. We explicate these mechanisms subsequently.

#### Narrative

People achieve GSC by constructing a narrative to make sense of changes in self and life (Chandler et al., 2003; see also Bluck & Alea, 2008; Prebble et al., 2013). Narrative, then, binds self-relevant (i.e., motivationally significant) events at different temporal junctures and builds coherent selfhood with turning points and causal progressions that extend into the future (Hammack, 2008; McAdams, 2011a; McLean et al., 2007). As already stated, narrative contributes to GSC (Becker et al., 2018; see also Habermas & Köber, 2015a; McAdams, 1985). Crucially, the narrative may qualify as a mediator of the relation between nostalgia and GSC. Indeed, in nostalgizing, people typically express their recollections in narrative form (Sedikides et al., 2015b; Wildschut et al., 2006, 2018).

#### Associative links

People may also attain GSC by creating associative links (i.e., objects, thoughts, feelings, or actions) between their present and past selves (Becker et al., 2018). For example, they use valued possessions to feel closer to their past selves and hence preserve self-continuity, particularly at major life transitions (Habermas & Paha, 2002; Kroger & Adair, 2008). Furthermore, a certain associative link may have relevance to one’s future; for example, a coffee mug may not only remind one nostalgically of a friend but also reinforce the expectation of continued interactions with that friend. Associative links predict GSC (Becker et al., 2018). Importantly, associative links may mediate the relation between nostalgia and GSC. Consistent with this claim, when nostalgizing, individuals link the past (e.g., “When I look at my family photo on my desk”) with the present (“I smile”; Stephan et al., 2012) and likely with the future (“I expect to be smiling for years to come”; Cheung et al., 2020).

#### Stability

Finally, people achieve GSC, in part, by emphasizing a self that is stable over time and by denying or minimizing change (Chandler et al., 2003). Stability refers to the degree of sameness of identity and its resilience to change across time, be it past, present, or future (Chandler et al., 2003; Ross, 1989). As mentioned, stability predicts GSC (Becker et al., 2018; see also Sokol & Serper, 2019). Critically, stability may qualify as a mediator of the relation between nostalgia and GSC. When nostalgizing, individuals bring to mind past selves that are central and authentic (Lasaleta & Loveland, 2019; Stephan et al., 2012) and hence likely enduring or stable (Markus, 1977; Sedikides, 1995).

### Overview

In Study 1, we set the stage by testing whether nostalgia is positively related to GSC. In Study 2, we examined whether the relation between nostalgia and GSC is mediated by narrative, associative links, and stability. In Study 3, we tested whether these patterns held after controlling for rumination, a correlate of nostalgia. We also varied whether identity aspects were chosen by participants or assigned by the experimenter. In Study 4, we probed the causal effect of nostalgia on GSC as mediated by the three bases of GSC.

Studies 1 to 4 were exploratory. However, Study 5 was confirmatory and preregistered at https://aspredicted.org/blind.php?x=ns2yg7. Here, we tested the hypotheses that (a) nostalgia elevates GSC and (b) narrative mediates the effect of nostalgia on GSC. We provide the stimulus materials and report ancillary analyses in the supplemental material. We obtained ethical approval from the authors’ institution. We deposited the data and code for the analyses at the Open Science Framework: https://osf.io/k6j2f/?view_only=4de49119c7c04b48bfd52750da59c4e3.

## Study 1

In Study 1, we examined whether dispositional nostalgia is related positively to GSC.

### Method

#### Participants

We tested 254 Amazon Mechanical Turk workers residing in the United States (149 men, 104 women, and 1 unreported; *M*_age_ = 35.29, *SD*_age_ = 12.03) for payment (US$0.50). We planned to recruit at least 250 participants (Schönbrodt & Perugini, 2013).

#### Materials and procedure

First, we assessed dispositional nostalgia. Participants completed the 7-item Southampton Nostalgia Scale (Barrett et al., 2010; Routledge et al., 2008). Three items measure the extent to which participants find nostalgia valuable, important, or significant (e.g., “How valuable is nostalgia for you?”; 1 = *not at all*, 7 = *very much*). Another four items measure proneness to nostalgia (e.g., “How prone are you to feeling nostalgic?”; 1 = *not at all*, 7 = *very much*) or frequency of nostalgic engagement (e.g., “Generally speaking, how often do you bring to mind nostalgic experiences?”; 1 = *very rarely*, 7 = *very frequently*). We averaged responses to form an index (α = .93, *M* = 4.58, *SD* = 1.13).

Then, we assessed GSC by adapting Becker et al.’s (2018) measure. Participants listed five identity aspects that best represented who they were and rated the degree to which each aspect contributed to GSC: “To what extent does [identity aspect] make you feel that your past, present, and future are connected?” (0 = *not at all*, 10 = *extremely*). We averaged responses to form a GSC index (Intraclass Correlation [ICC] *=* .79, *M* = 7.54, *SD* = 1.91).^1^ At the conclusion of the testing session, we collected demographic information (as we did in all studies).

### Results and Discussion

We found a positive correlation between dispositional nostalgia and the GSC index, *r*(252) = .41, *p* < .001. Nostalgia is related to higher GSC.

## Study 2

In Study 2, we examined whether narrative, associative links, and stability mediate the relation between dispositional nostalgia and GSC. We conducted this study in Korea. We intended to assess the replicability and cross-cultural generalizability of Study 1 results and to extend them.

### Method

#### Participants

We tested online 250 undergraduate and graduate student volunteers from various Korean universities (135 women, 94 men, 9 preferred not-to-answer, and 12 unreported; *M*_age_ = 21.84, *SD*_age_ = 2.65). Of the participants, 156 were affiliated with Sogang University, 58 with Yonsei University, 22 with Kyungbook University, 10 with Soonchunhyang University, and four were unreported. Similar to Study 1, we set to recruit at least 250 participants.

#### Materials and procedure

All materials were translated into Korean and back-translated into English by a committee of bilinguals (Brislin, 1970). First, participants completed the Southampton Nostalgia Scale (α = .91, *M* = 4.74, *SD* = 1.13). Then, they completed measures of GSC, narrative, associative links, and stability (Becker et al., 2018). Specifically, they listed seven identity aspects that best represented who they were and subsequently rated (0 = *not at all*, 10 = *extremely*) the degree to which they regarded each identity aspect as continuous (“To what extent does [identity aspect] make you feel that your past, present, and future are connected?”; ICC = .76, *M* = 6.91, *SD* = 1.50), narrative (“How much does [identity aspect] make you think of your life as a story?”; ICC = .75, *M* = 7.03, *SD* = 1.53), associatively linked (“How much does [identity aspect] remind you of the past?”; ICC = .68, *M* = 6.76, *SD* = 1.64), and stable (“To what extent is [identity aspect] stable and unchanging?”; ICC = .73, *M* = 6.91, *SD* = 1.50).

### Results and Discussion

#### Preliminary analysis

We carried out correlational analyses to test relations among nostalgia, stability, narrative, associative links, and GSC at individual and identity levels. At the individual level of analysis (*N* = 250), nostalgia was positively related to narrative, associative links, stability, and GSC. Narrative, associative links, and stability were also positively related to GSC (Table 1). We obtained similar results at the identity level of analysis (*N* = 1,750).

**Table 1.** Individual-Level (Below Diagonal) and Identity-Level (Above Diagonal) Zero-Order Correlations Among Variables in Study 2.

Measure	1	2	3	4	5
1. Nostalgia	—	—	—	—	—
2. Narrative	.226**	—	.500***	.497***	.516***
3. Associative links	.360***	.528***	—	.293***	.384***
4. Stability	.212**	.617***	.399***	—	.391***
5. Self-continuity	.300***	.578***	.486***	.472***	—

*Note.* The level of nostalgia varied between individuals but not between identity aspects rated by the same individual. We therefore report only individual-level correlations involving nostalgia.

** *p* < .01. ***p < .001.

#### Parallel mediation analysis

**Figure 1.**
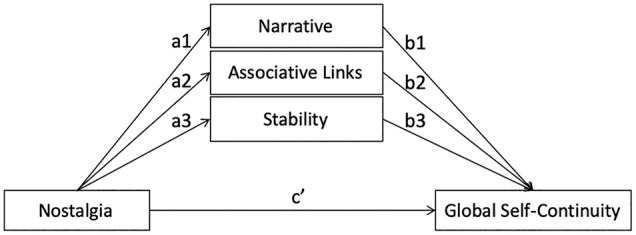
Parallel mediation path from nostalgia to global self-continuity through narrative, associative links, and stability.

We used the PROCESS macro (Hayes, 2018; Model 4, 10,000 bootstraps) to test a parallel mediation model (Figure 1). This individual-level, parallel mediation model tests the role of each mediator in the nostalgia–GSC link while controlling for the other two mediators. As per Table 2, nostalgia was positively related to narrative, associative links, and stability (a paths). The direct effect of nostalgia on GSC (controlling for the mediators) was also significant (c′ path, Table 2). In addition, narrative, associative links, and stability were positively related to GSC, controlling for nostalgia (b paths, Table 2). The results revealed significant indirect effects (denoted as *ab*) through narrative, *ab* = 0.11, *SE* = 0.04, 95% confidence interval (CI) = [0.038, 0.194], and associative links, *ab* = 0.09, *SE* = 0.04, 95% CI = [0.028, 0.171], but not through stability, *ab* = 0.04, *SE* = 0.02, 95% CI = [−0.002, 0.091]. Narrative and associative links independently mediated the relation between nostalgia and GSC, whereas stability did not.^2^

**Table 2.** Parallel Mediation Analysis in Studies 2 to 5.

Path	*B*	*SE*	*t*	*p*	LLCI	ULCI
Study 2						
a1	0.30	0.08	3.65	<.001	0.140	0.468
a2	0.52	0.09	6.08	<.001	0.352	0.690
a3	0.31	0.09	3.41	<.001	0.129	0.482
b1	0.35	0.07	5.24	<.001	0.218	0.481
b2	0.18	0.06	3.23	.001	0.070	0.289
b3	0.14	0.06	2.37	.018	0.023	0.251
c′	0.15	0.07	2.21	.028	0.017	0.292
Study 3						
a1	0.36	0.10	3.60	<.001	0.165	0.563
a2	0.57	0.09	5.98	<.001	0.380	0.754
a3	0.12	0.08	1.45	.150	–0.045	0.294
b1	0.31	0.05	6.58	<.001	0.216	0.401
b2	0.36	0.05	7.08	<.001	0.258	0.457
b3	0.16	0.05	3.06	.002	0.057	0.264
c′	−0.01	0.07	−0.20	.842	−0.153	0.125
Study 4						
a1	0.56	0.28	1.99	.048	0.005	1.105
a2	−0.18	0.26	−0.70	.483	−0.703	0.334
a3	0.46	0.23	1.99	.049	0.003	0.910
b1	0.35	0.07	5.15	<.001	0.216	0.484
b2	0.10	0.07	1.41	.160	−0.042	0.251
b3	0.13	0.08	1.73	.085	−0.019	0.283
c′	0.22	0.21	1.08	.283	−0.187	0.635
Study 5						
a1	1.32	0.26	5.04	<.001	0.802	1.836
a2	1.54	0.24	6.30	<.001	1.055	2.019
a3	0.02	0.25	0.09	.926	−0.479	0.526
b1	0.34	0.09	3.82	<.001	0.164	0.515
b2	0.08	0.09	0.88	.380	−0.104	0.271
b3	0.22	0.08	2.72	.007	0.060	0.379
c′	0.27	0.28	0.97	.335	−0.283	0.824

*Note*. All paths are between-person estimates. LLCI = lower limit confidence interval; ULCI = upper limit confidence interval. Paths: a1 = nostalgia to narrative, a2 = nostalgia to associative links, a3 = nostalgia to stability, b1 = narrative to continuity, b2 = associative links to continuity, b3 = stability to continuity, c′ = direct effect of nostalgia on continuity.

### Summary

We obtained a positive relation between nostalgia and GSC in a Korean sample, replicating the Study 1 results. Also, we replicated the Becker et al. (2018) finding that narrative, associative links, and stability predict higher GSC. More importantly, nostalgia predicted these three bases of GSC. Finally, two of the three bases—narrative and associative links—independently mediated the relation between nostalgia and GSC.

## Study 3

In Study 2, we instructed participants to choose seven identity aspects and, for each, rate the corresponding levels of GSC as well as the three bases of GSC (i.e., narrative, associative links, and stability). In Study 3, we manipulated whether identity aspects were chosen by participants versus assigned to them by the experimenter.^3^ We did so to test an alternative proposal. Dispositional nostalgia may conduce to selecting identities that afford high GSC; that is, routine nostalgizing may contribute to selecting those identities that satisfy the self-continuity motive. However, our argument has been that nostalgia contributes to increases in the degree of GSC that participants derive from their identities. If the alternative proposal is correct, then the positive relation between nostalgia and GSC should nullify when identities are assigned by the experimenter. In contrast, if our theoretical proposal is correct, then the positive relation between nostalgia and GSC should persist regardless of whether identities are freely chosen or assigned.

We offered another advancement in Study 3. Specifically, we scrutinized the role of rumination, a correlate of nostalgia. Rumination, “repetitively and passively focusing on symptoms of distress and on the possible causes and consequences of these symptoms” (Nolen-Hoeksema et al., 2008, p. 400), overlaps conceptually with nostalgia given that both constructs involve recruitment of memories for the purpose of current functioning. Yet, compared to ruminative memories, nostalgic memories more strongly serve the functions of intimacy maintenance (sustaining symbolic proximity to close others), teach/inform (sharing insights about life or oneself with others), as well as problem-solving/self-regard (using effective problem-solving strategies to guide present actions for one’s benefit), and more weakly serve the function of bitterness revival (rekindling resentments; Cheung et al., 2018; Jiang et al., 2021). We asked whether rumination might account for the relation of nostalgia with the putative mediators and GSC.

Finally, we randomized (separately for each participant) the measurement order of GSC, narrative, associative links, and stability. By doing so, we were able to rule out a possible order effect in Study 2, namely, that reflecting on GSC contributed to identity aspects being perceived as more narrative, associatively linked, and stable.

### Method

#### Participants

We tested via Prolific Academic 250 U.K.-based participants (177 women, 70 men, and three other; *M*_age_ = 36.14, *SD_age_* = 12.08) who reported English as their first language—a study requirement. We paid them £1.50 ($1.83). As in Studies 1 and 2, we set the minimum sample size at 250.

#### Materials and procedure

We assessed rumination first. Participants completed the 22-item Ruminative Responses Scale (Treynor et al., 2003). Sample items are: “Think about a recent situation, wishing it had gone better” and “Go someplace alone to think about your feelings” (1 = *almost never*, 4 = *almost always*; α = .94, *M* = 2.40, *SD* = 0.68). Then, we assessed dispositional nostalgia. Participants completed the Southampton Nostalgia Scale (α = .94, *M* = 4.41, *SD* = 1.38). Finally, we collected measures pertaining to narrative (ICC = .86, *M* = 6.34, *SD* = 2.25), associative links (ICC = .82, *M* = 6.14, *SD* = 2.20), stability (ICC = .78, *M* = 7.02, *SD* = 1.88), and GSC (ICC = .83, *M* = 6.66, *SD* = 2.01). We made several changes. First, participants listed five (as opposed to seven) identity aspects. Given the added variable (i.e., rumination), we attempted to simplify the procedure and reduce the possibility of participant fatigue. Second, half of the participants (identity choice condition) freely chose the five identity aspects, whereas the other half (identity assigned condition) were assigned five identity aspects. These were friendly, hardworking, happy, dependable, and resourceful. We selected them based on prior findings documenting that these identity aspects are considered generally important by participants (Sedikides, 1993; Sedikides et al., 2007; Sedikides & Green, 2000).

### Results and Discussion

#### Preliminary analysis

We conducted correlational analyses to examine relations among nostalgia, stability, narrative, associative links, and GSC at individual and identity levels. At the individual level of analysis (*N* = 250), nostalgia was positively related to narrative, associative links, and GSC but—contrary to Study 2—not stability. Narrative, associative links, and stability were positively related to GSC. We found similar results at the identity level of analysis (*N* = 1,270). Rumination was unrelated to all variables, except stability, with which it was positively related (Table 3).

**Table 3.** Individual-Level (Below Diagonal) and Identity-Level (Above Diagonal) Zero-Order Correlations Among Variables in Study 3.

Measure	1	2	3	4	5	6
1. Nostalgia	—	—	—	—	—	—
2. Rumination	.171**	—	—	—	—	—
3. Narrative	.223***	.051	—	.405***	.290***	.506***
4. Associative link	.355***	.062	.480***	—	.239***	.472***
5. Stability	.091	−.169**	.310***	.342***	—	.345***
6. Self-continuity	.221***	−.033	.580***	.607***	.391***	—

*Note.* Levels of nostalgia and rumination varied between individuals but not between identity aspects rated by the same individual. We therefore report only individual-level correlations involving nostalgia and rumination.

* *p* < .05. **p < .01. ***p < .001.

#### Parallel mediation analysis

We focused on the indirect effects of nostalgia on self-continuity through narrative, associative links, and stability via Hayes’ (2018) PROCESS macro (Model 4, 10,000 bootstraps) in testing an individual level, parallel mediation model. Nostalgia was positively related to narrative and associative links but, contrary to Study 2, was unrelated to stability (a paths, Table 2). The direct effect of nostalgia on GSC (controlling for the mediators) was not significant (c′ path, Table 2). The mediators predicted GSC, controlling for nostalgia (b paths, Table 2). Consistent with Study 2, results revealed significant indirect effects through narrative, *ab* = 0.11, *SE* = 0.04, 95% CI = [0.045, 0.191], and associative links, *ab* = 0.20, *SE* = 0.05, 95% CI = [0.116, 0.305], but not through stability, *ab* = 0.02, *SE* = 0.02, 95% CI = [−0.008, 0.058]. The pattern of indirect effects remained intact after controlling rumination for narrative, *ab* = 0.11, *SE* = 0.04, 95% CI = [0.042, 0.195]; for associative links, *ab* = 0.20, *SE* = 0.05, 95% CI = [0.118, 0.306]; and for stability, *ab* = 0.03, *SE* = 0.02, 95% CI = [−0.001, 0.063].

#### Moderated mediation analyses

We ran moderated mediation analyses (PROCESS macro, Model 8, 10,000 bootstraps; Hayes, 2018) to test whether the choice manipulation (0 = *identity choice*, 1 = *identity assigned*) moderated any indirect effects of nostalgia on GSC through narrative, associative links, and stability. The index of moderated mediation (IMM) was not significant for narrative, IMM = 0.04, *SE* = 0.07, 95% CI = [−0.090, 0.184]; associative links, IMM = −0.03, *SE* = 0.07, 95% CI = [−0.173, 0.120]; or stability, IMM = 0.04, *SE* = 0.03, 95% CI = [−0.015, 0.106].^4^ This nonsignificant moderated mediation is consistent with the possibility that nostalgia contributes to increases in the degree of GSC that participants derive from their identities. That is, individuals high on dispositional nostalgia derive more GSC, via narrative and associative links, even from assigned identity aspects.

#### Summary

We obtained a positive relation between nostalgia and GSC, in replication of Studies 1 and 2. Also, consistent with the Study 2 results, Study 3 revealed that nostalgia predicted higher GSC through narrative and associative links. Moreover, the same pattern emerged whether participants freely chose or were assigned identities. This finding aligns with our proposal that nostalgia increases the degree of GSC that participants derive from their identities.

## Study 4

In Studies 1 to 3, we obtained a positive relation between nostalgia and GSC through narrative and associative links. However, due to the correlational designs, we are unable to draw causal inferences. Does nostalgia augment the bases of GSC, and, in turn, do the latter predict increases in GSC? Therefore, in Study 4, we induced nostalgia experimentally, assessed all three bases of GSC as putative mediators, and assessed GSC as the outcome.

### Method

#### Participants

We tested 174 U.K.-based participants who reported English as their first language, through Prolific Academic (127 women, 46 men, and 1 other; *M*_age_ = 35.91, *SD*_age_ = 14.25) and paid them £1.00 ($1.22). Based on relevant research (Sedikides et al., 2015a), we estimated medium effect size and aimed to recruit a minimum of 140 participants to achieve power (1 - *β*) = .90 at α = .05. We randomly assigned them to the nostalgia (*n* = 85) or control (*n* = 89) condition.

#### Materials and procedure

First, we manipulated nostalgia (vs. control) with the Event Reflection Task. Participants in the nostalgia condition recalled a nostalgic event, described as “feeling sentimental about a fond and valued memory from one’s personal past,” listed four relevant keywords and wrote about how it made them feel. Participants in the control condition recalled a “past event that is ordinary, normal, and every day—that is, events that you experience on a regular basis,” listed four pertinent keywords, and wrote how it made them feel. Then, they completed a three-item manipulation check (Hepper et al., 2012; Wildschut et al., 2006). A sample item is: “Right now, I am feeling quite nostalgic” (1 = *strongly disagree*, 7 = *strongly agree*; α = .98, *M* = 4.05, *SD* = 1.57). Finally, they completed measures of narrative (ICC = .80, *M* = 7.27, *SD* = 1.85), associative links (ICC = .61, *M* = 6.74, *SD* = 1.73), and stability (ICC = .70, *M* = 7.82, *SD* = 1.53) pertaining to five participant-generated identity aspects, along with a measure of GSC (ICC = .67, *M* = 7.54, *SD* = 1.58), as in Study 3.

### Results and Discussion

#### Nostalgia manipulation

We conducted a one-way ANOVA to examine the effects of nostalgia on the manipulation check, the three bases of GSC, and GSC (Table 4). Participants in the nostalgia condition reported feeling more nostalgic than those in the control condition, *F*(1, 172) = 93.81, *p* < .001, η_p_^2^ = .353. Furthermore, participants in the nostalgia (vs. control) condition reported higher narrative, *F*(1, 172) = 3.97, *p* = .048, η_p_^2^ = .023, stability, *F*(1, 172) = 3.95, *p* = .049, η_p_^2^ = .022, and GSC, *F*(1, 172) = 3.71, *p* = .056, η_p_^2^ = .021, but not higher associative links, *F*(1, 172) = 0.50, *p* = .483, η_p_^2^ = .003.

**Table 4.** Descriptive Statistics for the Nostalgia and Control Conditions in Study 4 (Individual Level).

Dependent variable	Nostalgia condition	Control condition
	*M* (*SD*)	*M* (*SD*)
Manipulation Check (Felt Nostalgia)	5.00 (1.05)	3.14 (1.44)
Narrative	7.56 (1.63)	7.00 (2.02)
Associative links	6.64 (1.62)	6.82 (1.83)
Stability	8.06 (1.33)	7.60 (1.68)
Self-continuity	7.77 (1.49)	7.31 (1.65)

#### Correlational analysis

We carried out correlational analyses to test relations among the manipulation check (felt nostalgia), narrative, associative links, stability, and GSC at individual and identity levels. At the individual level of analysis (*N* = 174), felt nostalgia was positively related to narrative, stability, and GSC but not associative links. Narrative, associative links, and stability were also positively related to GSC (Table 5). We obtained similar results at the identity level of analysis (*N* = 870).

**Table 5.** Individual-Level (Below Diagonal) and Identity-Level (Above Diagonal) Zero-Order Correlations Among Variables in Study 4.

Measure	1	2	3	4	5
1. Felt Nostalgia	—	—	—	—	—
2. Narrative	.239**	—	.420***	.301***	.508***
3. Associative links	.107	.551***	—	.322***	.432***
4. Stability	.201**	.417***	.446***	—	.361***
5. Self-continuity	.227**	.536***	.393***	.360***	—

*Note.* Felt nostalgia varied between individuals but not between identity aspects rated by the same individual. We therefore report only individual-level correlations involving felt nostalgia.

* *p* < .05. **p < .01. ***p < .001.

#### Parallel mediation analysis

We used Hayes’ (2018) PROCESS macro (Model 4, 10,000 bootstraps) to test the parallel mediation model (Figure 1). Consistently, nostalgia (compared to control) led to higher stability and narrative but had no influence on associative links (a paths, Table 2). The direct effect of nostalgia on GSC (controlling for the mediators) was not significant (c′ path, Table 2). Moreover, only narrative was significantly positively related to GSC, controlling for the nostalgia manipulation (the association between stability and GSC was trending; b paths, Table 2). The results revealed a significant indirect effect through narrative, *ab* = 0.19, *SE* = 0.12, 95% CI = [0.005, 0.457], but not through associative links, *ab* = −0.02, *SE* = 0.04, 95% CI = [−0.130, 0.050], or stability, *ab* = 0.06, *SE* = 0.06, 95% CI = [−0.010, 0.205]. While narrative mediated the effect of nostalgia on GSC, associative links and stability did not do so.

#### Summary

Nostalgia increased GSC. This causal relation replicates conceptually the correlational relation between nostalgia and GSC reported in Studies 1 to 3. Moreover, the effect of nostalgia on GSC was transmitted by narrative only, whereas in correlational Studies 2 and 3 both narrative and associative links had emerged as mediators.

## Study 5

Given the partial inconsistencies in the correlational (Studies 2 and 3) and experimental (Study 4) findings in regard to mediators, we opted to test the replicability of Study 4 findings in preregistered Study 5. We hypothesized that nostalgia would increase GSC, and it would do so via narrative but not via associative links or stability.

We addressed two additional issues. First, in Study 4, we had assumed that the effect of nostalgic reverie would spread out to identify aspects and GSC. In Study 5, we tethered directly participants’ memories (nostalgic vs. ordinary) to the putative mediators and outcome; that is, we examined the extent to which the nostalgic memory per se would strengthen the narrative, associative links, stability, and consequently GSC. Relatedly, we operationalized stability in terms of the extent to which identity aspects contributed to perceptions of self as being unchanging rather than the extent to which the identity aspects were unchanging (a feature of all prior studies). Second, it is possible that the null mediational effect of stability in Study 4 was partially due to an instructional particularity. In particular, the wording of instructions in the control condition (i.e., to recall an ordinary event “that you experience on a regular basis”) may have elevated stability to a level similar to that of the experimental condition. We proceeded to test this possibility.

### Method

#### Participants

We recruited via Prolific Academic 148 U.K.-based participants (having planned to recruit 140, as in Study 4), and remunerated them with £0.70 ($0.85). We excluded two participants because they answered “no” to the question: “Did you pay attention to this survey while completing? Please be honest with your answer. Your answer will not affect your completion approval.” (Their inclusion did not change the results.) We randomly allocated the remaining 146 participants (89 women, 56 men, and 1 other; *M*_age_ = 38.95, *SD*_age_ = 12.12) to the nostalgia (*n* = 74) or control (*n* = 72) condition.

#### Materials and procedure

We manipulated nostalgia with a slightly modified version of the Event Reflection Task. Although we left the nostalgia condition instructions essentially unaltered, we made a minor change to the control condition. Specifically, we asked control condition participants to “bring to mind an ordinary event in your life. . ., a past event that is ordinary” but did not further instruct them to recall an event they experienced “on a regular basis.” Next, all participants listed four applicable keywords, described how the recalled event made them feel, and completed the same manipulation check as in Study 4 (α = .98, *M* = 4.28, *SD* = 1.51). Finally, they responded (1 = *not at all*, 7 = *very much*) to measures of narrative (“How much does this memory make you think of your life as a story?”; *M* = 4.86, *SD* = 1.71), associative links (“How much does this memory remind you of your past self or identity?”, *M* = 4.92, *SD* = 1.66), stability (“To what extent does this memory make you see yourself as stable and unchanging?”; *M* = 4.14, *SD* = 1.53), and GSC (“To what extent does this memory make you feel that your past, present, and future are connected?”; *M* = 4.90, *SD* = 1.68). Although we randomized the order of the three mediators separately for each participant, we always assessed GSC last.

### Results and Discussion

#### Nostalgia manipulation

We examine the effects of nostalgia on the manipulation check, the three bases of GSC, and GSC via one-way ANOVAs (Table 6). Participants in the nostalgia condition reported feeling more nostalgic than their control condition counterparts, *F*(1, 144) = 44.18, *p* < .001, η_p_^2^ = .235. Moreover, participants in the nostalgia (vs. control) condition reported higher narrative, *F*(1, 144) = 25.42, *p* < .001, η_p_^2^ = .150, associative links, *F*(1, 144) = 39.67, *p* < .001, η_p_^2^ = .216, and GSC, *F*(1, 144) = 9.98, *p* = .002, η_p_^2^ = .065, albeit not higher stability, *F*(1, 144) = 0.01, *p* = .926, η_p_^2^ < .001 (despite the wording changes in the control condition).

**Table 6.** Descriptive Statistics for the Nostalgia and Control Conditions in Study 5.

Dependent variable	Nostalgia condition	Control condition
	*M* (*SD*)	*M* (*SD*)
Manipulation check (Felt Nostalgia)	5.00 (0.97)	3.54 (1.62)
Narrative	5.51 (1.46)	4.19 (1.70)
Associative links	5.68 (1.14)	4.14 (1.76)
Stability	4.15 (1.58)	4.13 (1.49)
Self-continuity	5.32 (1.66)	4.47 (1.60)

#### Correlation analysis

We carried out correlational analyses to examine relations among felt nostalgia (the manipulation check), narrative, associative links, stability, and GSC. Felt nostalgia was positively related to narrative, associative links, stability, and GSC. Narrative, associative links, and stability were also positively related to GSC (Table 7).

**Table 7.** Zero-Order Correlations Among Variables in Study 5.

Measure	1	2	3	4	5
1. Felt Nostalgia	—				
2. Narrative	.513***	—			
3. Associative links	.554***	.570***	—		
4. Stability	.183*	.105	.059	—	
5. Self-continuity	.311***	.445***	.329***	.241**	—

* *p* < .05. **p < .01. ***p < .001.

#### Parallel mediation analysis

We used the PROCESS macro (Hayes, 2018; Model 4, 10,000 bootstraps) to test a parallel mediation model (Figure 1). Nostalgia (compared to control) led to higher narrative and associative links but had no influence on stability (a paths, Table 2). The direct effect of nostalgia on GSC (controlling for the mediators) was not significant (c′ path, Table 2). Moreover, narrative and stability were positively related to GSC but associative links was not related with GSC, controlling for the nostalgia manipulation (b paths, Table 2). The indirect effect was significant through narrative, *ab* = 0.45, *SE* = 0.16, 95% CI = [0.153, 0.789], but not associative links, *ab* = 0.13, *SE* = 0.17, 95% CI = [−0.183, 0.467], or stability, *ab* = 0.005, *SE* = 0.06, 95% CI = [−0.122, 0.123].

#### Summary

As hypothesized, and replicating the Study 4 findings, nostalgic (vs. control) participants reported higher GSC via narrative but not via associative links or stability.

## General Discussion

The literature has indicated that nostalgia is positively related to past-present self-continuity (Zou et al., 2018) and fosters past-present self-continuity (Sedikides et al., 2015a, 2016c; Wildschut et al., 2019). We advanced this literature in two ways. First, we examined whether nostalgia is positively related to, and fosters, GSC. Second, we examined whether nostalgia’s relation to GSC is transmitted via three bases of GSC: narrative, associative links, and stability.

### Summary of Findings

In five studies, we established a naturalistic (Studies 1–3) and causal (Studies 4 and 5) relation between nostalgia and GSC. We further found that nostalgia conduces to gains in the amount of GSC that participants derive from their identities, even when these identities were assigned by the experimenter (Study 3). In addition, we specified that nostalgia relates to, or elevates, GSC chiefly through narrative (Studies 2–5) and less so through associative links (Studies 2 and 3) but not through stability. Finally, we ruled out the relevance of a nostalgia correlate, rumination (Study 3).

### Theoretical Implications

Following assessment of their dispositional nostalgia (Studies 1–3) or induction of nostalgia (Study 4), participants listed important aspects of their identity (e.g., relational roles, social roles, and traits), and rated these aspects for the degree of GSC. Identity aspects were generated by participants in Studies 1 to 4, but in Study 3, half of them were assigned these aspects by the experimenter. We wanted to know whether being handed out nomothetically important (Sedikides, 1993, 1995) identity aspects makes a difference. Might this externality demotivate participants (Ryan & Deci, 2017)? If nostalgia conduced to increases in their motivation to generate their own identities and elevate their GSC, then the assignment of identity aspects by the experimenter would block nostalgia’s motivational potency. This is not what we found. Nostalgia’s strength in conducing to bases of GSC and GSC was equivalent regardless of whether the identity aspects were self-generated or externally provided. These findings are preliminary and need to be replicated with different methods. Yet, the findings offer a glimpse at underlying processes. Due to its reservoir of meaningful memories (Sedikides & Wildschut, 2018), nostalgia can activate vital bases of GSC, which in turn strengthen GSC (Studies 2–5). The findings reinforce the notion that nostalgia is a self-relevant emotion (Van Tilburg et al., 2018; Vess et al., 2012; Wildschut et al., 2006).

Narrative emerged as the key mediator. Narrative serves to conjoin events from one’s past, adding coherence to it (Becker et al., 2018; McAdams, 2001b; McLean et al., 2020). Furthermore, narrative helps to conjoin possible events in one’s future (Landau et al., 2014; McAdams, 1985). As nostalgic memories help to construe life in a narrative form (Sedikides et al., 2015b; Wildschut et al., 2006, 2018), it is reasonable that narrative emerged as the principal construct to tether nostalgia to GSC. Narrative may not only contribute to GSC but also recruit to counter self-discontinuity (i.e., a sense of disconnection among one’s past, present, and future self; cf. Sedikides et al., 2008, 2015a). Some preliminary correlational and developmental data point to that possibility (Habermas & Köber, 2015b), which awaits corroboration by experimental tests.

Associative links, but not stability, played a mediational role in correlational Studies 2 to 3. In experimental Study 4, the nostalgia induction (i.e., Event Reflection Task) had no discernible impact on associative links, but it did increase stability. In experimental Study 5, the nostalgia induction had no discernible impact on stability, but it increased associative links. How can these empirical discrepancies be reconciled?

Associative links appeared to be relevant in correlational, but not experimental, designs. This empirical discrepancy opens up two possibilities. First, alternative nostalgia inductions might boost the mediational potency of associative links. Specifically, this construct has been theorized to operate at a more intuitive or less explicit level than narrative or stability (Becker et al., 2018). Nostalgia inductions, then, may evoke associative links via an implicit sense of closeness (as when nostalgia is induced by music, song lyrics, or scents; Cheung et al., 2013; Reid et al., 2015) or via vividness (as when nostalgia in induced by mementos; Fairley et al., 2018; Kalnikaitė & Whittaker, 2011). Second, the long-term effects of dispositional nostalgia may diverge to some extent from the short-term effects of state or induced nostalgia. Future research might address these possibilities.

Stability has been theorized to be the central strategy that people use to attain GSC (Chandler et al., 2003) and lay theories concur (Quoidbach et al., 2013). Our findings supported the relation between stability and GSC. In each pertinent study, stability was significantly correlated with GSC, and this relation remained essentially intact even when controlling for nostalgia and the other bases of GSC (b3 paths, Table 2). However, the relation between nostalgia and stability was more tenuous (a3 paths, Table 2). Nostalgia can entail a contrast between past and present (Iyer & Jetten, 2011; Wildschut et al., 2019); in such cases, nostalgia highlights change rather than stability.

### Limitations and Future Research Directions

Our work has several limitations. To begin, we did not address the downstream implications of nostalgia’s effect on GSC. Relevant literature has shown that present-to-past self-continuity, as induced by nostalgia, confers well-being benefits and in particular subjective vitality (i.e., feeling alert and energetic; Sedikides et al., 2016c) and meaning in life (Van Tilburg et al., 2019a). Future research would do well to test whether nostalgia-induced GSC confers similar benefits. Also, we assessed GSC and its bases with single items. Although such a practice has been validated by Becker et al. (2018), and relevant findings are replicated here, follow-up investigations could index each construct with multiple items and validate more extensively these measures.

Hong et al. (2020) recently reported that nostalgia increased GSC through a facet of holistic thinking termed interactional causality. This refers to the assumption that multiple causes interact to influence an outcome (e.g., “Everything in the universe is somehow related to each other”; Choi et al., 2007). Interactional causality may be independent of narrative or part of it. Future research would do well to address this issue.

Our work could be complemented and, indeed, enhanced by the narrative research method (Habermas & Köber, 2015b; Jennings & McLean, 2013). This method assigns secondary importance to self-reports of remembering and recounting (i.e., a sense of or perception of the past) while assigning primary importance to actual narratives (e.g., life stories) that participants relay. These life stories, then, are analyzed in accord with one’s empirical objectives. In our case, participants’ stories pertaining to the putative mediators (i.e., narrative, associative links, stability) would be entered as a mediator of the relation between nostalgia and GSC. A developmental perspective would further expand the research agenda (Arnett, 2000; McAdams & Cox, 2010).

### Coda

Five studies converged in illustrating that nostalgia is positively related to and fosters GSC, primarily through narrative. The findings clarified the psychological relevance of nostalgia, widened the conceptual scope of self-continuity (from past-to-present to global), and explicated the mechanisms by which nostalgia relates to or promotes GSC.

## Supplemental Material

sj-docx-1-psp-10.1177_01461672211024889 – Supplemental material for How Does Nostalgia Conduce to Global Self-Continuity? The Roles of Identity Narrative, Associative Links, and Stability Click here for additional data file. Supplemental material, sj-docx-1-psp-10.1177_01461672211024889 for How Does Nostalgia Conduce to Global Self-Continuity? The Roles of Identity Narrative, Associative Links, and Stability by Emily K. Hong, Constantine Sedikides and Tim Wildschut in Personality and Social Psychology Bulletin

## Notes

1 We report the mean and standard deviation for within-subjects variables (i.e., self-continuity, narrative, associative links, and stability) at the aggregated, individual level.

2 In Studies 2 to 4, identity aspects were nested within individuals. Accordingly, we also conducted multilevel mediation analyses. These analyses produced essentially identical results to the individual-level mediation analyses. We report the multilevel mediation analyses for Studies 2 to 4 in the supplemental material, Ancillary Analyses.

3 Assigned identities, like chosen identities, reflect aspects of the self-concept (Robinson & Sedikides, 2009, 2020)

4 We further examined indirect effects in each condition. For narrative, the indirect effect was significant for the identity choice condition, *ab* = 0.09, *SE* = 0.05, 95% CI = [0.004, 0.197], and the identity assigned condition, *ab* = 0.13, *SE* = 0.05, 95% CI = [0.044, 0.243]. For associative links, the indirect effect was significant for the identity choice condition, *ab* = 0.22, *SE* = 0.06, 95% CI = [0.107, 0.352], and the identity assigned condition, *ab* = 0.19, *SE* = 0.06, 95% CI = [0.088, 0.324]. For stability, the indirect effect was not significant for the identity choice condition, *ab* = 0.003, *SE* = 0.02, 95% CI = [−0.031, 0.040], nor the identity assigned condition, *ab* = 0.04, *SE* = 0.03, 95% CI = [−0.002, 0.097].
