# Supplementary material for: How Does Nostalgia Conduce to Global Self-Continuity? The Roles of Identity Narrative, Associative Links, and Stability
Source: Pers Soc Psychol Bull. 2021 Jun 21;48(5):735–49. doi: 10.1177/01461672211024889 (PMC9066684; doi:10.1177/01461672211024889)
Supplement: sj-docx-1-psp-10.1177_01461672211024889 – Supplemental material for How Does Nostalgia Conduce to Global Self-Continuity? The Roles of Identity Narrative, Associative Links, and Stability [file sj-docx-1-psp-10.1177_01461672211024889.docx]

**SUPPLEMENTARY MATERIALS**

**for**

**How Does Nostalgia Conduce to Global Self-Continuity?**

**The Roles of Identity Narrative, Associative Links, and Stability**

**Southampton Nostalgia Scale Used in Studies 1-3**

According to the Oxford Dictionary, ‘nostalgia’ is defined as a ‘sentimental longing for the past.’

1. How valuable is nostalgia for you?

| Not at all |  |  |  |  |  | Very much |
| --- | --- | --- | --- | --- | --- | --- |
| 1 | 2 | 3 | 4 | 5 | 6 | 7 |

2. How important is it for you to bring to mind nostalgic experiences?

| Not at all |  |  |  |  |  | Very much |
| --- | --- | --- | --- | --- | --- | --- |
| 1 | 2 | 3 | 4 | 5 | 6 | 7 |

3. How significant is it for you to feel nostalgic?

| Not at all |  |  |  |  |  | Very much |
| --- | --- | --- | --- | --- | --- | --- |
| 1 | 2 | 3 | 4 | 5 | 6 | 7 |

4. How prone are you to feeling nostalgic?

| Not at all |  |  |  |  |  | Very much |
| --- | --- | --- | --- | --- | --- | --- |
| 1 | 2 | 3 | 4 | 5 | 6 | 7 |

5. How often do you experience nostalgia?

| Not at all |  |  |  |  |  | Very much |
| --- | --- | --- | --- | --- | --- | --- |
| 1 | 2 | 3 | 4 | 5 | 6 | 7 |

6. Generally speaking, how often do you bring to mind nostalgic experiences?

| Not at all |  |  |  |  |  | Very much |
| --- | --- | --- | --- | --- | --- | --- |
| 1 | 2 | 3 | 4 | 5 | 6 | 7 |

7. Specifically, how often do you bring to mind nostalgic experiences? (Please check one.)

_____ At least once a day

_____ Three to four times a week

_____ Approximately twice a week

_____ Approximately once a week

_____ Once or twice a month

_____ Once every couple of months

_____ Once or twice a year

**Ruminative Responses Scale Used in Study 3**

People think and do many different things when they feel depressed. Please read each of the items below and indicate whether you almost never, sometimes, often, or almost always think or do each one when you feel down, sad, or depressed. Please indicate what you generally do, not what you think you should do.

1 = *almost never* 2 = *sometimes* 3 = *often* 4 = *almost always*

1. think about how alone you feel
2. think “I won’t be able to do my job if I don’t snap out of this”
3. think about your feelings of fatigue and achiness
4. think about how hard it is to concentrate
5. think “What am I doing to deserve this?”
6. think about how passive and unmotivated you feel.
7. analyze recent events to try to understand why you are depressed
8. think about how you don’t seem to feel anything anymore
9. think “Why can’t I get going?”
10. think “Why do I always react this way?”
11. go away by yourself and think about why you feel this way
12. write down what you are thinking about and analyze it
13. think about a recent situation, wishing it had gone better
14. think “I won’t be able to concentrate if I keep feeling this way.”
15. think “Why do I have problems other people don’t have?”
16. think “Why can’t I handle things better?”
17. think about how sad you feel.
18. think about all your shortcomings, failings, faults, mistakes
19. think about how you don’t feel up to doing anything
20. analyze your personality to try to understand why you are depressed
21. go someplace alone to think about your feelings
22. think about how angry you are with yourself

**Identity Aspects Measure Used in Studies 1-4**

*Studies 2: 7 things about yourself*

*Studies 1, 3, and 4: 5 things about your self*

In the spaces below, please write down 5 things about yourself. You can write your answers as they occur to you without worrying about the order, but together they should summarize the image you have of who you are. You can write anything you think describes you well. Your answers might include social groups or categories you belong to, personal relationships with others, as well as characteristics of yourself as an individual. Some may be things that other people know about, others may be your private thoughts about yourself. Some things you may see as relatively important, and others less so. Some may be things you are relatively happy about, and others less so.

1. _____________________
2. _____________________
3. _____________________
4. _____________________
5. _____________________

To what extent does each of these things make you feel that your past, present, and future are connected?

|  | **Not**  **at all** | |  |  | |  | |  | |  | |  | |  | |  | |  | | **Very much** | |  |
| --- | --- | --- | --- | --- | --- | --- | --- | --- | --- | --- | --- | --- | --- | --- | --- | --- | --- | --- | --- | --- | --- | --- |
| **A.** | 0 | 1 | | | 2 | | 3 | | 4 | | 5 | | 6 | | 7 | | 8 | | 9 | | 10 | |
| **B.** | 0 | 1 | | | 2 | | 3 | | 4 | | 5 | | 6 | | 7 | | 8 | | 9 | | 10 | |
| **C.** | 0 | 1 | | | 2 | | 3 | | 4 | | 5 | | 6 | | 7 | | 8 | | 9 | | 10 | |
| **D.** | 0 | 1 | | | 2 | | 3 | | 4 | | 5 | | 6 | | 7 | | 8 | | 9 | | 10 | |
| **E.** | 0 | 1 | | | 2 | | 3 | | 4 | | 5 | | 6 | | 7 | | 8 | | 9 | | 10 | |

How much does each of these things make you think of your life as a story?

|  | **Not**  **at all** | |  |  | |  | |  | |  | |  | |  | |  | |  | | **Very much** | |  |
| --- | --- | --- | --- | --- | --- | --- | --- | --- | --- | --- | --- | --- | --- | --- | --- | --- | --- | --- | --- | --- | --- | --- |
| **A.** | 0 | 1 | | | 2 | | 3 | | 4 | | 5 | | 6 | | 7 | | 8 | | 9 | | 10 | |
| **B.** | 0 | 1 | | | 2 | | 3 | | 4 | | 5 | | 6 | | 7 | | 8 | | 9 | | 10 | |
| **C.** | 0 | 1 | | | 2 | | 3 | | 4 | | 5 | | 6 | | 7 | | 8 | | 9 | | 10 | |
| **D.** | 0 | 1 | | | 2 | | 3 | | 4 | | 5 | | 6 | | 7 | | 8 | | 9 | | 10 | |
| **E.** | 0 | 1 | | | 2 | | 3 | | 4 | | 5 | | 6 | | 7 | | 8 | | 9 | | 10 | |

How much does each of these things remind of you of the past?

|  | **Not**  **at all** | |  |  | |  | |  | |  | |  | |  | |  | |  | | **Very much** | |  |
| --- | --- | --- | --- | --- | --- | --- | --- | --- | --- | --- | --- | --- | --- | --- | --- | --- | --- | --- | --- | --- | --- | --- |
| **A.** | 0 | 1 | | | 2 | | 3 | | 4 | | 5 | | 6 | | 7 | | 8 | | 9 | | 10 | |
| **B.** | 0 | 1 | | | 2 | | 3 | | 4 | | 5 | | 6 | | 7 | | 8 | | 9 | | 10 | |
| **C.** | 0 | 1 | | | 2 | | 3 | | 4 | | 5 | | 6 | | 7 | | 8 | | 9 | | 10 | |
| **D.** | 0 | 1 | | | 2 | | 3 | | 4 | | 5 | | 6 | | 7 | | 8 | | 9 | | 10 | |
| **E.** | 0 | 1 | | | 2 | | 3 | | 4 | | 5 | | 6 | | 7 | | 8 | | 9 | | 10 | |

To what extent is each of these things stable and unchanging?

|  | **Not**  **at all** | |  |  | |  | |  | |  | |  | |  | |  | |  | | **Very much** | |  |
| --- | --- | --- | --- | --- | --- | --- | --- | --- | --- | --- | --- | --- | --- | --- | --- | --- | --- | --- | --- | --- | --- | --- |
| **A.** | 0 | 1 | | | 2 | | 3 | | 4 | | 5 | | 6 | | 7 | | 8 | | 9 | | 10 | |
| **B.** | 0 | 1 | | | 2 | | 3 | | 4 | | 5 | | 6 | | 7 | | 8 | | 9 | | 10 | |
| **C.** | 0 | 1 | | | 2 | | 3 | | 4 | | 5 | | 6 | | 7 | | 8 | | 9 | | 10 | |
| **D.** | 0 | 1 | | | 2 | | 3 | | 4 | | 5 | | 6 | | 7 | | 8 | | 9 | | 10 | |
| **E.** | 0 | 1 | | | 2 | | 3 | | 4 | | 5 | | 6 | | 7 | | 8 | | 9 | | 10 | |

**Study 3: Identity Assigned Condition**

In the spaces below, you will see five things about yourself. Together, they summarize an image of who you are. Some may be things that other people know about you; others may be your private thoughts about yourself. Some things you may see as relatively important, and others less so. Some may be things you are relatively happy about and others less so.

1. Friendly
2. Hardworking
3. Happy
4. Dependable
5. Resourceful

To what extent does each of these things make you feel that your past, present, and future are connected?

|  | **Not**  **at all** | |  |  | |  | |  | |  | |  | |  | |  | |  | | **Very much** | |  |
| --- | --- | --- | --- | --- | --- | --- | --- | --- | --- | --- | --- | --- | --- | --- | --- | --- | --- | --- | --- | --- | --- | --- |
| Friendly | 0 | 1 | | | 2 | | 3 | | 4 | | 5 | | 6 | | 7 | | 8 | | 9 | | 10 | |
| Hardworking | 0 | 1 | | | 2 | | 3 | | 4 | | 5 | | 6 | | 7 | | 8 | | 9 | | 10 | |
| Happy | 0 | 1 | | | 2 | | 3 | | 4 | | 5 | | 6 | | 7 | | 8 | | 9 | | 10 | |
| Dependable | 0 | 1 | | | 2 | | 3 | | 4 | | 5 | | 6 | | 7 | | 8 | | 9 | | 10 | |
| Resourceful | 0 | 1 | | | 2 | | 3 | | 4 | | 5 | | 6 | | 7 | | 8 | | 9 | | 10 | |

How much does each of these things make you think of your life as a story?

|  | **Not**  **at all** | |  |  | |  | |  | |  | |  | |  | |  | |  | | **Very much** | |  |
| --- | --- | --- | --- | --- | --- | --- | --- | --- | --- | --- | --- | --- | --- | --- | --- | --- | --- | --- | --- | --- | --- | --- |
| Friendly | 0 | 1 | | | 2 | | 3 | | 4 | | 5 | | 6 | | 7 | | 8 | | 9 | | 10 | |
| Hardworking | 0 | 1 | | | 2 | | 3 | | 4 | | 5 | | 6 | | 7 | | 8 | | 9 | | 10 | |
| Happy | 0 | 1 | | | 2 | | 3 | | 4 | | 5 | | 6 | | 7 | | 8 | | 9 | | 10 | |
| Dependable | 0 | 1 | | | 2 | | 3 | | 4 | | 5 | | 6 | | 7 | | 8 | | 9 | | 10 | |
| Resourceful | 0 | 1 | | | 2 | | 3 | | 4 | | 5 | | 6 | | 7 | | 8 | | 9 | | 10 | |

How much does each of these things remind of you of the past?

|  | **Not**  **at all** | |  |  | |  | |  | |  | |  | |  | |  | |  | | **Very much** | |  |
| --- | --- | --- | --- | --- | --- | --- | --- | --- | --- | --- | --- | --- | --- | --- | --- | --- | --- | --- | --- | --- | --- | --- |
| Friendly | 0 | 1 | | | 2 | | 3 | | 4 | | 5 | | 6 | | 7 | | 8 | | 9 | | 10 | |
| Hardworking | 0 | 1 | | | 2 | | 3 | | 4 | | 5 | | 6 | | 7 | | 8 | | 9 | | 10 | |
| Happy | 0 | 1 | | | 2 | | 3 | | 4 | | 5 | | 6 | | 7 | | 8 | | 9 | | 10 | |
| Dependable | 0 | 1 | | | 2 | | 3 | | 4 | | 5 | | 6 | | 7 | | 8 | | 9 | | 10 | |
| Resourceful | 0 | 1 | | | 2 | | 3 | | 4 | | 5 | | 6 | | 7 | | 8 | | 9 | | 10 | |

To what extent is each of these things stable and unchanging?

|  | **Not**  **at all** | |  |  | |  | |  | |  | |  | |  | |  | |  | | **Very much** | |  |
| --- | --- | --- | --- | --- | --- | --- | --- | --- | --- | --- | --- | --- | --- | --- | --- | --- | --- | --- | --- | --- | --- | --- |
| Friendly | 0 | 1 | | | 2 | | 3 | | 4 | | 5 | | 6 | | 7 | | 8 | | 9 | | 10 | |
| Hardworking | 0 | 1 | | | 2 | | 3 | | 4 | | 5 | | 6 | | 7 | | 8 | | 9 | | 10 | |
| Happy | 0 | 1 | | | 2 | | 3 | | 4 | | 5 | | 6 | | 7 | | 8 | | 9 | | 10 | |
| Dependable | 0 | 1 | | | 2 | | 3 | | 4 | | 5 | | 6 | | 7 | | 8 | | 9 | | 10 | |
| Resourceful | 0 | 1 | | | 2 | | 3 | | 4 | | 5 | | 6 | | 7 | | 8 | | 9 | | 10 | |

**Mediators and Self-Continuity Measures in Study 5**

We would like you to answer a few questions about your memory of the nostalgic (ordinary) event.

How much does this memory make you think of your life as a story? (1 = *not at all*, 7 = *very much*)

How much does this memory remind you of your past self or identity? (1 = *not at all*, 7 = *very much*)

To what extent does this memory make you see yourself as stable and unchanging? (1 = *not at all*, 7 = *very much*)

To what extent does this memory make you feel that your past, present, and future are connected? (1 = *not at all*, 7 = *very much*)

**Event Reflection Task Used in Study 4**

*Nostalgia Condition*

Nostalgia is defined as a sentimental longing for one’s past or as feeling sentimental about a fond and valued memory from one’s personal past (e.g., childhood, close relationships, significant events). Now, please think of a nostalgia event in your life. Specifically, try to think of a past event that makes you feel most nostalgic. Bring this nostalgic experience to mind. Immerse yourself in this nostalgic experience for two minutes, and think about how it makes you feel.

Please write down four keywords relevant to this nostalgic event (i.e., words that describe the nostalgic experience).

Keywords that describe my nostalgic experience:

(Next page)

Using the space provided below, for the next five minutes, we would now like you to write about the nostalgic memory. Immerse yourself into the thoughts and feelings associated with this memory. Describe this nostalgic memory and how it makes you feel warm and sentimental. Be as thorough as possible in describing how you are feeling.

*Control Condition*

Please think of an ordinary event in your life. Specifically, try to think of a past event that is ordinary, normal, and everyday—that is, events that you experience on a regular basis (e.g., getting on a bus, shopping at the supermarket, watching television). Bring this ordinary experience to mind, immerse yourself in the ordinary experience for two minutes, and think about how it makes you feel. Please write down four keywords relevant to this ordinary event (i.e., words that describe the ordinary experience).

Keywords that describe my ordinary experience:

(Next page)

Using the space provided below, for the next five minutes, we would now like you to write about the ordinary, normal, and everyday event. Immerse yourself into this experience. Describe this recent event and what it makes you think about. Be as thorough as possible in describing what you are thinking.

**Event Reflection Task Used in Study 5**

*Nostalgia Condition*

According to the Oxford Dictionary, ‘nostalgia’ is defined as a ‘sentimental longing for one’s past.’ Please think of a nostalgic event in your life. Specifically, try to think of a past event that makes you feel most nostalgic. Bring this nostalgic experience to mind. Immerse yourself in the nostalgic experience. How does it make you feel? Please write down four keywords relevant to this nostalgic event (i.e., words that describe the experience).

Keywords that describe my nostalgic experience:

(Next page)

Using the space provided below, for the next few minutes, we would like you to write about the nostalgic event. Immerse yourself into this nostalgic experience. Describe the experience and how it makes you feel.

*Control Condition*

Please bring to mind an ordinary event in your life. Specifically, try to think of a past event that is ordinary. Bring this ordinary experience to mind. Immerse yourself in the ordinary experience. How does it make you feel? Please write down four keywords relevant to this ordinary event (i.e., words that describe the experience).

Keywords that describe my ordinary experience:

(Next page)

Using the space provided below, for the next few minutes, we would like you to write about this ordinary event. Immerse yourself into this ordinary experience. Describe the experience and how it makes you feel.

**Manipulation Check Used in Studies 4 and 5**

The following statements refer to how you feel right now. Please indicate your agreement or disagreement by placing a number in the blank space preceding each statement. The number should be anywhere from 1 to 6, according to the following scale.

| 1 | 2 | 3 | 4 | 5 | 6 |
| --- | --- | --- | --- | --- | --- |
| Strongly disagree | Moderately disagree | Slightly disagree | Slightly  agree | Moderately agree | Strongly  agree |

___ Right now, I am feeling quite nostalgic.

___ Right now, I am having nostalgic feelings

___ I feel nostalgic at the moment.

**ANCILLARY ANALYSES**

**Data-Analytic Approach**

For Studies 2-4, the identity aspects pertinent to the dependent measure (i.e., GSC) and mediators (i.e., narrative, associative links, stability) were nested within individual participants. Therefore, in addition to the individual-level mediation analyses reported in the main text, we also carried out multilevel mediation analyses. The multilevel models included two levels. Level 1 units were identity aspects nested within participants, and Level 2 units were participants. To account for dependence among responses by the same individual, we estimated the slopes and intercepts as random effects (Bryk & Raudenbush, 1992; Singer, 1998).

**Multilevel Parallel Mediation Analysis**

We used Hayes and Rockwood’s (2017) MLmed macro (REML estimation with 10,000 Monte Carlo samples) to estimate parameters for a 2–1–1 parallel mediation model. This aggregated model tests the role of each mediator in the association between nostalgia and GSC, while controlling for the other two.

***Study 2***

The indirect effects (denoted as *ab*) through narrative, *ab* = 0.11, *SE* = 0.04, 95% Monte Carlo Confidence Interval (CI) = [0.044, 0.182], associative links, *ab* = 0.09, *SE* = 0.03, 95% CI = [0.035, 0.165], and stability, *ab* = 0.04, *SE* = 0.02, 95% CI = [0.006, 0.092] were significant. The significant indirect via stability in this multilevel mediation analysis stands in contrast to the nonsignificant indirect via stability in the individual-level mediation analysis. This was the only discrepancy between the multilevel and individual-level analyses. It is noteworthy, however, that the individual-level analyses revealed both a significant *a* path and a significant *b* path (see Table 2, main text). This means that the indirect effect via stability was also significant in the individual-level analyses if one adopts the joint-significance criterion advocated by Yzerbyt et al. (2018). In this case, there are no discrepancies between the multilevel and individual-level mediation analyses.

***Study 3***

The indirect effects through narrative, *ab* = 0.11, *SE* = 0.04, 95% CI = [0.049, 0.186], and associative links, *ab* = 0.20, *SE* = 0.04, 95% CI = [0.122, 0.298] were significant, but the indirect effect through stability was not, *ab* = 0.02, *SE* = 0.02, 95% CI = [-0.007, 0.056]. The pattern of indirect effects remained intact after controlling for rumination: *ab* = 0.11, *SE* = 0.04, 95% CI = [0.046, 0.191] for narrative; *ab* = 0.20, *SE* = 0.05, 95% CI = [0.123, 0.299] for associative links; and *ab* = 0.03, *SE* = 0.02, 95% CI = [-0.0003, 0.063] for stability.

We ran a multilevel moderated mediation analysis with the MLmed macro, to test whether the choice manipulation (0 = *identity choice*, 1 = *identity assigned*) moderated any indirect effects of nostalgia on GSC through narrative, associative links, and stability. The index of moderated mediation (IMM) was not significant for narrative, IMM = -0.01, 95% CI = [-0.039, 0.015], associative links, IMM = -0.01, 95% CI = [-0.043, 0.024], or stability, IMM = 0.02, 95% CI = [-0.003, 0.044].

***Study 4***

The indirect effect through narrative, *ab* = 0.19, *SE* = 0.11, 95% CI = [0.006, 0.414] was significant, but the indirect effects through associative links, *ab* = -0.02, *SE* = 0.04, 95% CI = [-0.104, 0.043], and stability, *ab* = 0.06, *SE* = 0.05, 95% CI = [-0.012, 0.175], were not.

**References**

Bryk, A.S., & Raudenbush, S.W. (1992). *Hierarchical linear models: Applications and data analysis methods*. Sage.

Hayes, A.F., & Rockwood, N.J. (2017). Regression-based statistical mediation and moderation analysis in clinical research: Observations, recommendations, and implementation. *Behaviour Research and Therapy*, *98*, 39–57. https://doi.org/10.1016/j.brat.2016.11.001

Pinheiro, J., Bates, D., DebRoy, S., Sarkar, D., & R Core Team (2020). nlme: Linear and Nonlinear Mixed Effects Models. R package version 3.1-150. [https://CRAN.R-project.org/package=nlme](https://eur03.safelinks.protection.outlook.com/?url=https%3A%2F%2Fcran.r-project.org%2Fpackage%3Dnlme&data=04%7C01%7CC.Sedikides%40soton.ac.uk%7Ce6d0fb774a3d493f347f08d88fc63c7c%7C4a5378f929f44d3ebe89669d03ada9d8%7C0%7C0%7C637417428867107482%7CUnknown%7CTWFpbGZsb3d8eyJWIjoiMC4wLjAwMDAiLCJQIjoiV2luMzIiLCJBTiI6Ik1haWwiLCJXVCI6Mn0%3D%7C1000&sdata=0elNGAe1g4YB0MjOtEG4RYlURMTnPeNzOYk1vFP8wbI%3D&reserved=0).

Singer, J.D. (1998). Using SAS PROC MIXED to fit multilevel models, hierarchical models, and individual growth models. *Journal of Educational and Behavioral Statistics*, *23*(6), 323–356. <https://doi.org/10.3102/10769986023004323>

Yzerbyt, V., Muller, D., Batailler, C., & Judd, C.M. (2018). New recommendations for testing indirect effects in mediational models: The need to report and test components paths. *Journal of Personality and Social Psychology, 115*(6), 929-943. https://doi.org/10.1037/pspa0000132
